# Seatbelt sign in a case of blunt abdominal trauma; what lies beneath it?

**DOI:** 10.1186/s12893-015-0108-z

**Published:** 2015-10-30

**Authors:** Michail G. Vailas, Demetrios Moris, Stamatios Orfanos, Chrysovalantis Vergadis, Alexandros Papalampros

**Affiliations:** First Surgical Department, Athens University School of Medicine, “Laiko” General Hospital, Agiou Thoma 17, Athens, 11527 Greece; Radiology Department, “Laiko” General Hospital, Agiou Thoma 17, Athens, 11527 Greece

**Keywords:** Blunt abdominal trauma, Colon injury, Hollow viscus injury

## Abstract

**Background:**

The reported incidence of hollow viscus injuries (HVI) in blunt trauma patients is approximately 1 %. The most common site of injury to the intestine in blunt abdominal trauma (BAT) is the small bowel followed by colon, with mesenteric injuries occurring three times more commonly than bowel injuries. Isolated colon injury is a rarely encountered condition. Clinical assessment alone in patients with suspected intestinal or mesenteric injury after blunt trauma is associated with unacceptable diagnostic delays.

**Case presentation:**

This is a case of a 31-year-old man, admitted to the emergency department after being the restrained driver, involved in a car accident. After initial resuscitation, focused assessment with sonography for trauma examination (FAST) was performed revealing a subhepatic mass, suspicious for intraperitoneal hematoma. A computed tomography scan (CT) that followed showed a hematoma of the mesocolon of the ascending colon with active extravasation of intravenous contrast material. An exploratory laparotomy was performed, hemoperitomeum was evacuated, and a subserosal hematoma of the cecum and ascending colon with areas of totally disrupted serosal wall was found. Hematoma of the adjacent mesocolon expanding to the root of mesenteric vessels was also noted. A right hemicolectomy along with primary ileocolonic anastomosis was performed. Patient’s recovery progressed uneventfully.

**Conclusion:**

Identifying an isolated traumatic injury to the bowel or mesentery after BAT can be a clinical challenge because of its subtle and nonspecific clinical findings; meeting that challenge may eventually lead to a delay in diagnosis and treatment with subsequent increase in associated morbidity and mortality. Isolated colon injury is a rare finding after blunt trauma and usually accompanied by other intra-abdominal organ injuries. Abdominal ‘seatbelt’ sign, ecchymosis of the abdominal wall, increasing abdominal pain and distension are all associated with HVI. However, the accuracy of these findings remains low. Diagnostic peritoneal lavage, ultrasound, CT and diagnostic laparoscopy are used to evaluate BAT. Although CT has become the main diagnostic tool for this type of injuries, there are few pathognomonic signs of colon injury on CT. Given the potential for devastating outcomes, prompt diagnosis and treatment is necessary and high clinical suspicion is required.

## Background

HVI are not common and occur in approximately 1 % of all blunt trauma patients. [1]. The most common site of injury to the intestine in BAT is the small bowel followed by colon with mesenteric injuries occurring three times more commonly than bowel injuries [2]. HVI are defined as a spectrum of bowel injury ranging from hematomas to full thickness defects in the walls of the stomach, small and large bowel. Mesenteric injuries may be isolated or accompanied by associated HVI. [1]. Bowel injuries that warrant surgical attention include full-thickness perforation, a seromuscular tear, and devascularized bowel. Significant mesenteric injuries include disruption of the mesentery, a mesenteric injury resulting in ischemic bowel, and active mesenteric bleeding [2].

The pathogenic mechanisms of HVI injury after BAT are substantially two, acting isolated or combined; compression forces and deceleration forces [3]. Early recognition is of superior importance because of the significant morbidity and mortality of this type of injuries [2, 4]. Diagnosis of bowel injury is associated with unacceptable diagnostic delays and is a difficult task in patients suffering from BAT [5]. Signs and symptoms often require a number of hours before clinical apparent peritonitis appears. Isolated colon injury following BAT is a rarely encountered condition since colon injury is usually accompanied by other intra-abdominal organ injuries. We report a case of an isolated traumatic injury of the cecum and ascending colon along with mesocolon injury after a motor vehicle accident.

## Case presentation

A 31-year-old man with no previous medical history was transported to the Emergency Department (ED) by ambulance on a spine board and with a cervical collar in place after a car accident. The patient was the restrained vehicle driver and the Emergency Medicine Services found him at the scene soon after a collision with a bus. His initial vital signs were notable for a blood pressure of 122/72 mm Hg, a heart rate of 122 beats/ min and a respiratory rate of 22 breaths/ min.

On arrival to the ED, the patient had a blood pressure of 115/65 mm Hg and a pulse of 128 beats/ min. Airway was patent with an oxygen saturation of 99 % through an oxygen mask. On primary survey, equal breath sounds were auscultated with no signs of associate underlying lung injury. Heart sounds were tachycardic but otherwise normal along with intact distal pulses in all extremities. Fluid resuscitation with normal saline was initiated in the field and continued through two large bore intravenous lines at both antecubital fossae. The patient’s GCS was calculated to be 15/15. Full body exposure revealed ‘seatbelt’ sign in the right lower quadrant of the abdomen and no other signs of traumatic injuries.

**Fig. 1:**
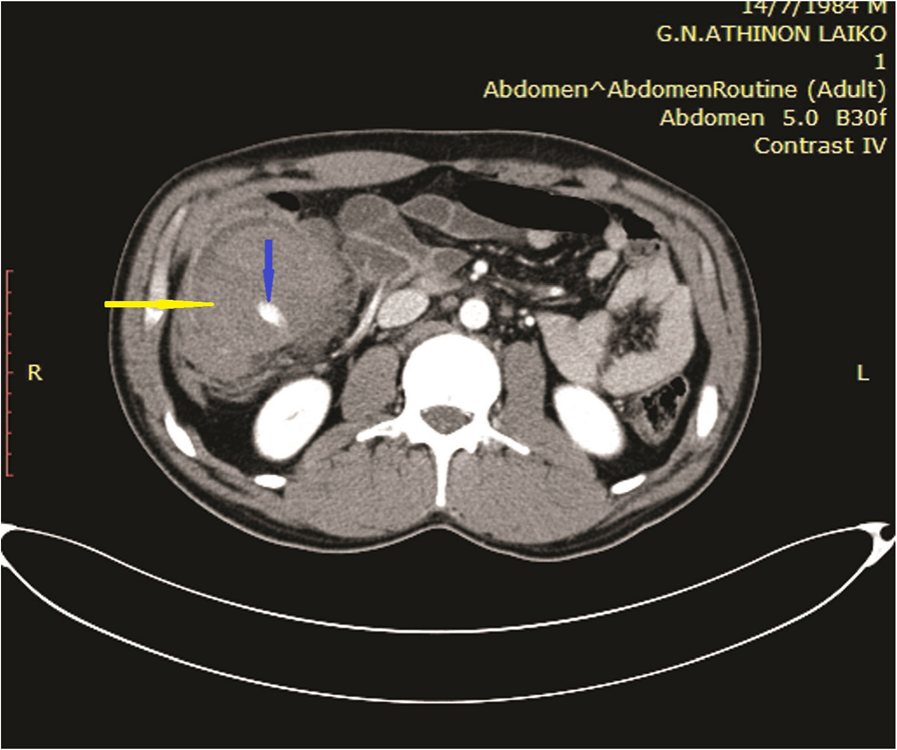
MDCT-axial image showing hematoma of the mesocolon of the ascending colon (yellow arrow). Active extravasation of intravenous contrast (blue arrow)

**Fig. 2:**
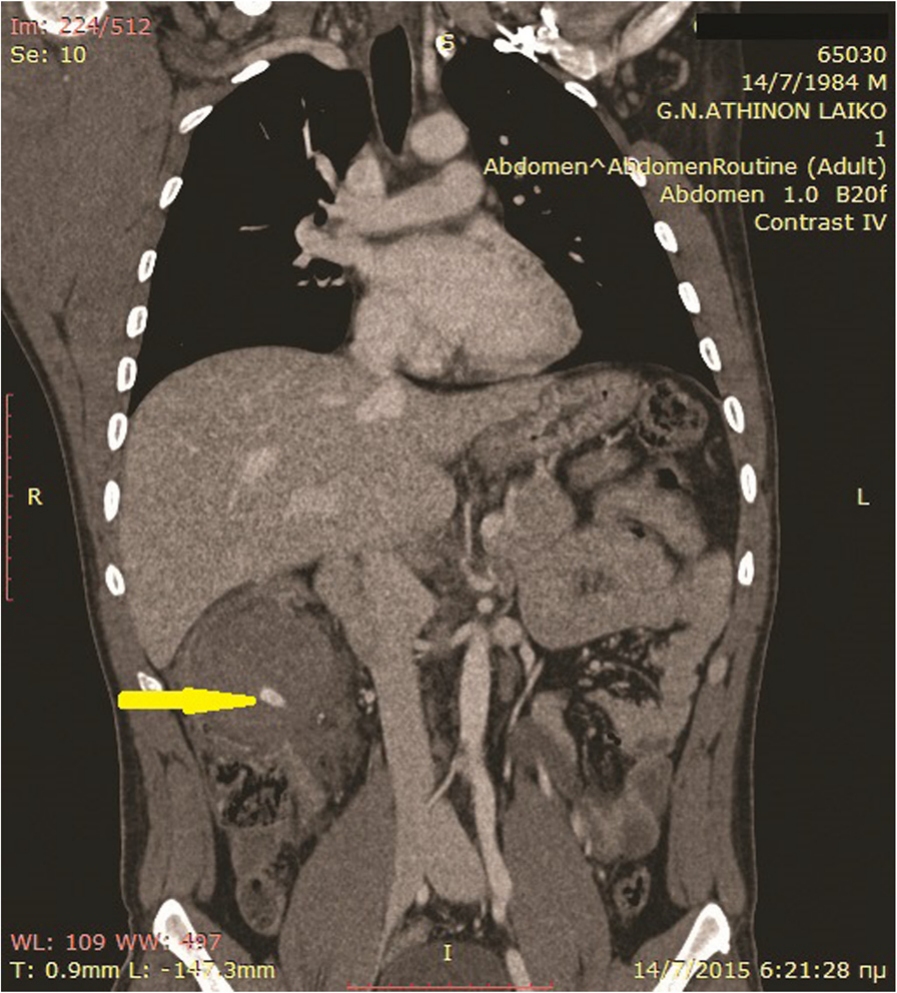
MDCT-coronal image showing active extravasation of intravenous contrast (yellow arrow)

On secondary survey, abrasions were noted on both upper extremities and there was abdominal discomfort with diffuse tenderness, guarding over the lower part of the abdomen with no signs of distension. The focused assessment with sonography for trauma examination (FAST) revealed a mixed-echogenicity subhepatic mass, suspicious for intraperitoneal hematoma. Chest, cervical spine and pelvic radiographs showed no abnormalities. Given his relative hemodynamic stability head to pelvis CT scanning was performed, which showed a hematoma of the mesocolon of the ascending colon with active extravasation of intravenous contrast material, originating from a branch of the right colic artery. A pseudoaneurysm of this arterial branch was also noted along with displacement of the ascending colon, resulted from the hematoma of the mesocolon (Figs. 1 and 2).

**Fig. 3:**
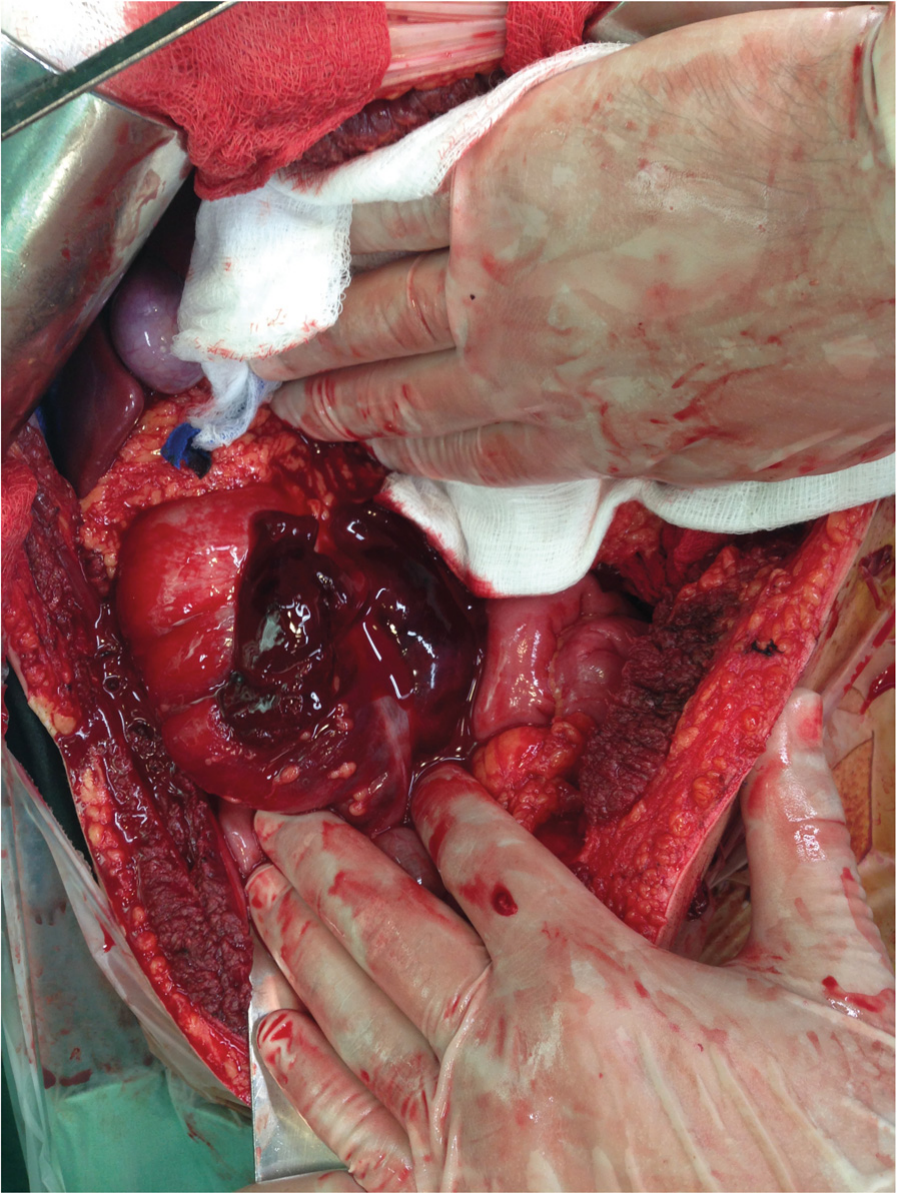
Subserosal hematoma of the cecum–disrupted serosal surface

**Fig. 4:**
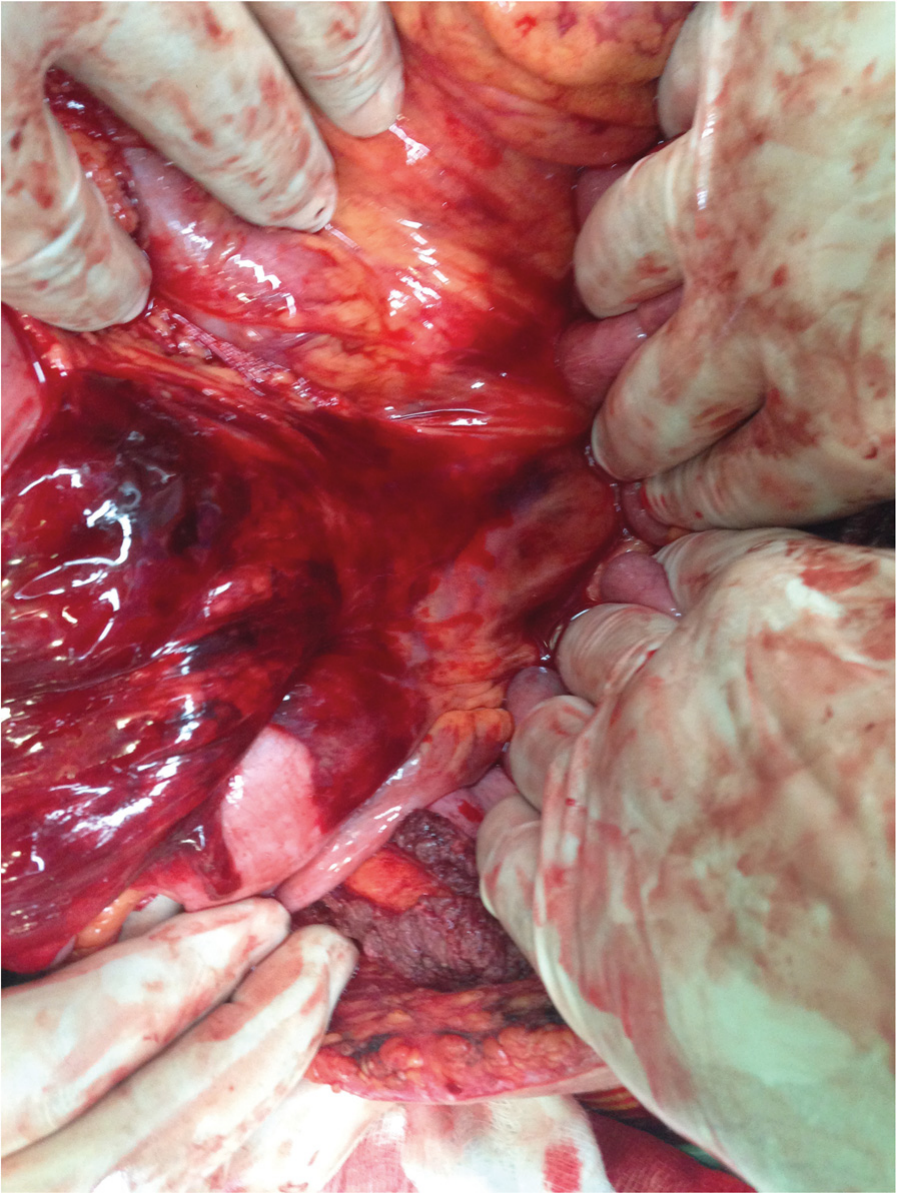
Expanding hematoma of the mesocolon

Diagnostic laparoscopy was a consideration as a management option but due to the deterioration of patient’s condition with increasing abdominal pain and overt peritoneal signs along with hemodynamic instability, the surgeon’s choice was to take the patient to the operating room for exploratory laparotomy with the fear that the induction of pneumoperitoneum would compromise venous flow return and could be easily fatal in this particular case. A median laparotomy was performed and an estimated 300 cc hemoperitoneum was evacuated. Intraoperative findings included a subserosal hematoma along the cecum and the adjacent segment of the ascending colon with areas of totally disrupted serosal wall (Fig. 3). There was also a v-shaped hematoma of mesocolon adjacent to injured bowel, expanding to the origin of the mesenteric vessels (Fig. 4). Intraoperative findings revealed no other traumatic injuries in abdominal structures. A standard right hemicolectomy along with primary ileotransverse colonic anastomosis was performed.

The patient was taken to the recovery room post-surgery in a stable condition and admitted to surgical intensive care unit. The postoperative course was uneventful and the patient was discharged on post-operative day seven without any problems.

## Discussion

In recent series, the reported incidence of HVI in blunt trauma patients is approximately 1 % [1]. In one study of 275,557 trauma patients, Watts et al. reported a 1.2 % rate of HVI in blunt trauma patients and 3.1 % in those receiving abdominal work-up [1]. Colon injury from BAT is uncommon, being diagnosed in about 0.5 % of all major blunt traumas. Most of the colonic injuries are ‘partial thickness’ (only 3 % undergoing laparotomy have ‘full thickness’ injuries) [3]. The ascending and descending colon, fixed, partially retroperitoneal segments, are exposed to more severe injuries compared to transverse and sigmoid colon, wrapped in their own mesocolon [3]. Isolated colon injury is a rarely encountered condition. Colon injury is usually accompanied by other intra-abdominal organ injuries, with the small intestine, spleen, liver, and pancreas being the leading areas [6]. Mesenteric injuries include a broad spectrum of traumatic findings from simple contusion to mesenteric avulsion. In the majority of the cases traumatic lesions to mesentery are isolated, rarely associated with lesions of the intestines [3].

BAT occurs most frequently in the context of motor vehicle accidents and is classified according to whether the primary mechanism of injury is related to a compression or deceleration force. Compression of abdominal organs occurs when there has been a direct blow on the abdomen or when there has been external compression against a fixed object such as steering wheels or seat belts. Seat-belt injury is caused by hyper-flexion around a lap belt which acts as a fulcrum. In these patients abdominal ‘seat-belt’ sign should aware the physician of an underlying HVI. Deceleration forces on the other hand cause stretching and linear shearing at interfaces between fixed and mobile parts of the gastrointestinal tract. As bowel loops course from their mesenteric attachments, thrombosis and mesenteric injuries occur [2, 6].

Since the introduction of the three-point seatbelt in the 1970s, fatalities from road traffic accidents have fallen by up to 60 %. Over the years, however, a well-defined injury pattern related to the use of seat belts has become the norm of presentation in patients involved in high energy impacts [7]. The patterns of injury, known as ‘seatbelt syndrome’, were originally described by Garrett and Braunstein in 1962, as linear ecchymosis of the abdominal wall following a motor vehicle accident [8]. The ‘seatbelt sign’ has recently expanded to the ‘seatbelt’ complex to describe a pattern of injury to the intestine, lumbar spine and other abdominal organs associated with the use of lap belts. Chest injuries, fractures of the sternum and ribs along with injury of the major vessels are also included in this complex [7, 8]. Chandler et al. (1997) [7] in a consecutive series of 117 patients injuries in motor vehicle accidents found that of their 11.9 % patients with a seatbelt sign, all had underlying bowel injuries and possibly other visceral abdominal injuries. Allen et al. (1998) [9] in a series of 19.621 patients (2,550 < or = 14 years old; 17,070 > 14 years old) found that 139 of 17,070 (0.8 %) adults had HVI compared with 27 of 2,550 (1 %) children. Among patients with abdominal wall ecchymosis, 13.5 % of children had HVI compared with 10.6 % of adults. Velmahos et al. (1999) [7] studied 410 patients wearing lap belts who were injured in road traffic accidents. 77 (12 %) had a seatbelt sign. Of these 77, 9 % suffered bruising of the neck, 32 % bruising of the chest, 40 % bruising of the abdomen and 19 % bruising across multiple areas on the body. 3 patients had myocardial contusion and 10 patients had intra-abdominal injuries (predominantly bowel and mesenteric lacerations) requiring laparotomy. There was a nearly four-fold increase in chest trauma (22.5 versus 6 %; P = 0.01) and a nearly eight-fold increase in intra-abdominal injury (23 versus 3 %; P < 0.0001) in the cohort restrained with seat belts as opposed to those without. Wotherspoon et al. (2001) [8] in a total of 99 patients with abdominal injuries after motor vehicle accidents found that the seatbelt sign was present in 60/99 cases. The proportion of intestinal injuries in patients with and without seatbelt sign were 9/60 and 0/39, respectively (P = 0.01). The prevalence of intestinal injuries in patients with seatbelt sign was 15 %. In a study involving a total of 147,985 children pediatric patients, the sensitivity, specificity, and positive and negative predictive values of abdominal wall bruising for significant intraabdominal injuries were 73.5 %, 98.8 %, 11.5 %, and 99.9 %, respectively [10]. Chidester et al. in a series of 331 pediatric patients have reported a sensitivity of 25 % and a specificity of 85 % of the seat belt sign in association with intra-abdominal injuries [11].

Physical examination is notoriously inaccurate in the diagnosis of BAT and requires a high index of clinical suspicion. Abdominal ‘seatbelt’ sign, ecchymosis of the abdominal wall, increasing abdominal pain and distension are all associated with HVI. However, the accuracy of these findings remains low [1]. DPL, US, CT and diagnostic laparoscopy are used to evaluate BAT [4]. Over the last decade, FAST examination and CT have minimized the role of DPL [1]. In the vast majority of trauma centers, multi-detector computer tomography (MDCT) is recognized as a primary tool in the diagnosis of traumatic injuries to the bowel and mesentery in the stable and semi-stable trauma patients. Few signs on CT are pathognomonic for HVI [3, 5]. Full thickness bowel injury includes the presence of extraluminal gas, intramural air, extraluminal oral contrast, extraluminal intestinal content and discontinuity of the bowel wall. In most of the cases, intraperitoneal fluid may be the sole finding of a significant bowel injury at the first CT evaluation [3]. Specific signs for mesenteric injuries on CT include avulsion of a meso resulting in ischemic changes of the loop, active bleeding and mesenteric hematoma [3].

Indications for laparotomy include haemodynamic instability with reasonable clinical suspicion of an underlying injury of the abdominal organs, positive abdominal signs on clinical examination such as peritonitis, increasing pain and distension, positive DPL, positive diagnostic imaging and abdominal finding by laparoscopy [4].

The lack of a widely accepted pathway for diagnosis of HVI creates difficulty for emergency physicians, resulting in increased morbidity and mortality rates. Delays, even as little as 6 to 8 hours, can lead to sepsis from abdominal contamination with intestinal contents after perforation, peritonitis, abscess formation, longer hospital stays and higher rates of acute respiratory distress syndrome [1]. Risk factors associated with adverse outcomes in colon trauma include hypotension or shock, interval of injury to operation, amount of fecal contamination, associated organ injury, number of transfusions and comorbidities [12].

Two grading scales have been developed to stratify injuries to the colon and their subsequent management. Flint et al. introduced a scale that can be summarized as follows: grade 1: minimal contamination, minimal delay to operation, no associated injuries, and minimal shock; grade 2: through-and-through perforations or lacerations with associated injuries; and grade 3: severe tissue loss, heavy contamination, and deep shock. The American Association for the Surgery of Trauma (AAST) developed the Colon Injury Scale (CIS): grade I, serosal injury; grade II, single wall injury; grade III, <25 % wall involvement; grade IV, >25 % wall involvement; grade V, circumferential colon wall, vascular injury or both [12]. Flint grades 1 and 2 and CIS grades I to III are considered nondestructive colon wounds and were found to be amenable to primary repair. Destructive wounds include Flint grade 3 or CIS graded IV and V injuries. The management of these injuries is less clear because they occur less frequently and less information is available. Patients with destructive injuries traditionally would be considered for fecal diversion, but the current trades have changed towards primary repair [12]. The 2001 landmark paper by Demetriades et al. prospectively compared outcomes between 197 colon-injured patients managed with primary repair and 100 colon-injured patients managed with fecal diversion. Management technique was not associated with adverse outcomes even in the sickest populations suffering from penetrating colon trauma [13].

As far as the management of abdominal emergencies is concerned, in recent years, the concept of ‘acute care laparoscopy’ is emerging, becoming a new discipline, aiming to join together the difficult issues of emergency surgery with the potential advantages of minimally invasive surgery. Apart from traditional exploratory laparotomy, emergency laparoscopy is now considered an alternative option for selected trauma cases. The only real contraindication to the use of laparoscopy in an emergency setting is in patients exhibiting hemodynamic instability and severe hemorrhagic or septic shock [14].

## Conclusions

Identifying an isolated traumatic injury to the bowel or mesentery after BAT can be a clinical challenge because of its subtle and nonspecific clinical findings; meeting that challenge may eventually lead to a delay in diagnosis and treatment with subsequent increase in associated morbidity and mortality. ‘Seatbelt’ sign is usually accompanied by underlying injuries of the abdominal organs. A high index of suspicion with a low threshold for appropriate diagnostic evaluation and/or surgical exploration is recommended for patients with ‘seatbelt sign’.

The most common site of injury to the intestines following BAT is the small bowel. Isolated colon injury is a rare finding after BAT and usually accompanied by other intra-abdominal organ injuries [6]. However, this was the case in our patient who was found to have a subserosal hematoma of the cecum and ascending colon along with an expanding hematoma of the adjacent mesocolon.

## Consent

Written informed consent was obtained from the patient for publication of this Case report and any accompanying images. A copy of the written consent is available for review by the Editor of this journal.

### Ethics

Research has been carried out within an appropriate ethical framework. However, ethics was not required for our study, since there is no such a committee approving studies in Greece.

## Abbreviations

BAT: blunt abdominal trauma
DPL: diagnostic peritoneal lavage
ED: emergency department
FAST: focused assessment with sonography for trauma examination
HVI: hollow viscus injuries
MDCT: multi-detector computer tomography

## References

[1] McStay C, Ringwelski A, Levy P, Legome E (2009). Hollow viscus injury. J Emerg Med.

[2] Khan I, Bew D, Elias DA, Lewis D, Meacock LM (2014). Mechanisms of injury and CT findings in bowel and mesenteric trauma. Clin Radiol.

[3] Iaselli F, Mazzei MA, Firetto C, D'Elia D, Squitieri NC, Biondetti PR (2015). Bowel and mesenteric injuries from blunt abdominal trauma: a review. Radiol Med.

[4] Zheng YX, Chen L, Tao SF, Song P, Xu SM (2007). Diagnosis and management of colonic injuries following blunt trauma. World J Gastroenterol.

[5] Scaglione M, De Lutio di Castelguidone E, Scialpi M, Merola S, Diettrich AI, Lombardo P (2004). Blunt trauma to the gastrointestinal tract and mesentery: is there a role for helical CT in the decision-making process?. Eur J Radiol.

[6] Ertugrul G, Coskun M, Sevinc M, Ertugrul F, Toydemir T (2012). Delayed presentation of a sigmoid colon injury following blunt abdominal trauma: a case report. J Med Case Rep.

[7] Biswas S, Adileh M, Almogy G, Bala M (2014). Abdominal injury patterns in patients with seatbelt signs requiring laparotomy. J Emerg Trauma Shock.

[8] Wotherspoon S, Chu K, Brown AF (2001). Abdominal injury and the seat-belt sign. Emerg Med (Fremantle).

[9] Allen GS, Moore FA, Cox CS, Wilson JT, Cohn JM, Duke JH (1998). Hollow visceral injury and blunt trauma. J Trauma.

[10] Lutz N, Nance ML, Kallan MJ, Arbogast KB, Durbin DR, Winston FK (2004). Incidence and clinical significance of abdominal wall bruising in restrained children involved in motor vehicle crashes. J Pediatr Surg.

[11] Chidester S, Rana A, Lowell W, Hayes J, Groner J (2009). Is the “seat belt sign” associated with serious abdominal injuries in pediatric trauma?. J Trauma.

[12] Maxwell RA, Fabian TC (2003). Current management of colon trauma. World J Surg.

[13] Hatch Q, Causey M, Martin M, Stoddard D, Johnson E, Maykel J (2013). Outcomes after colon trauma in the 21st century: an analysis of the U.S. National Trauma Data Bank. Surgery.

[14] Di Saverio S (2014). Emergency laparoscopy: a new emerging discipline for treating abdominal emergencies attempting to minimize costs and invasiveness and maximize outcomes and patients’ comfort. J Trauma Acute Care Surg.

